# [Corrigendum] Long non‑coding RNA AC245100.4 promotes prostate cancer tumorigenesis via the microRNA‑145‑5p/RBBP5 axis

**DOI:** 10.3892/or.2023.8499

**Published:** 2023-02-13

**Authors:** Hui Xie, Jiabin Zhao, Jiahui Wan, Jianing Zhao, Qianqian Wang, Xu Yang, Weiyu Yang, Ping Lin, Xiaoguang Yu

Oncol Rep 45: 619–629, 2021; DOI: 10.3892/or.2020.7894

Subsequently to the publication of the above article, an interested reader drew to the authors’ attention that, concerning the cell proliferation and migration assay data shown in Figs. 6D and 7B, there were a pair of panels showing overlapping data, such that the same data had apparently been selected to show the results from different experiments. Subsequently, the authors referred back to their original data, and identified further incorrectly assembled data panels in Figs. 3B and 7B.

The corrected versions of Fig. 3B (showing the correct data for the ‘AC245100.4 / PC3 / 0 h’ scratch-wound assay data panel), Fig. 6D (showing the correct data for the ‘PC3 / NC-mimic’ and ‘DU-145 / NC-inhibitor’ data panels) and Fig. 7D (showing the correct data for the ‘PC3 / 24 h / Inhibitor-miR-145-5p + siAC245100.4’ data panel) are shown on the subsequent pages. The authors regret the errors that were made during the preparation of the published figures, and confirm that these errors did not grossly affect the conclusions reported in the study. The authors are grateful to the Editor of *Oncology Reports* for allowing them the opportunity to publish a Corrigendum, and all the authors agree to this Corrigendum. Furthermore, they apologize to the readership for any inconvenience caused.

## Figures and Tables

**Figure 3.**
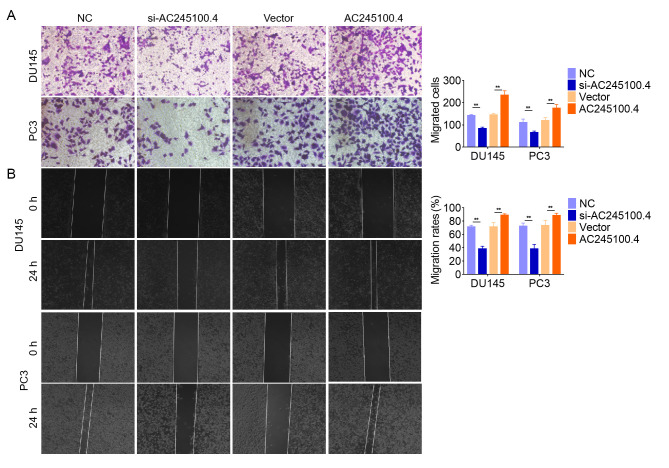
lncRNA AC245100.4 promotes PCa cell migration *in vitro*. (A) Effects of AC245100.4 on PCa cell migration were assessed using Transwell migration and (B) wound healing assays. Magnification, ×200. Data are presented as the mean ± SD of three independent experiments (n=3). **P<0.01. AC245100.4, lncRNA AC245100.4; lncRNA, long non-coding RNA; PCa, prostate cancer; NC, negative control; si, small interfering RNA.

**Figure 6.**
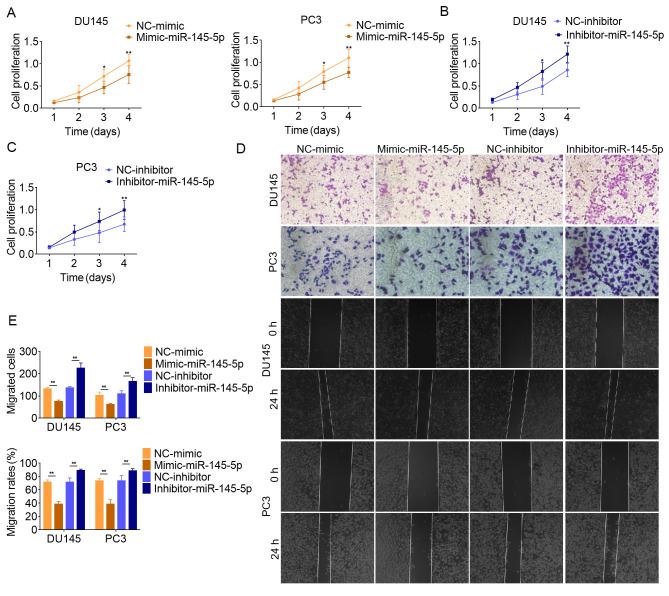
miR-145-5p serves as a tumor suppressor in PCa. Effects of miR-145-5p (A) mimic or (B and C) inhibitor on PCa cell proliferation were assessed using a Cell Counting Kit 8 assay. (D) Effects of miR-145-5p on PCa cell migration were assessed using Transwell migration and wound healing assays, and (E) the results were quantified. Magnification, ×200. Data are presented as the mean ± SD of three independent experiments (n=3). *P<0.05 and **P<0.01. miR-145-5p, microRNA-145-5p; PCa, prostate cancer; NC, negative control.

**Figure 7.**
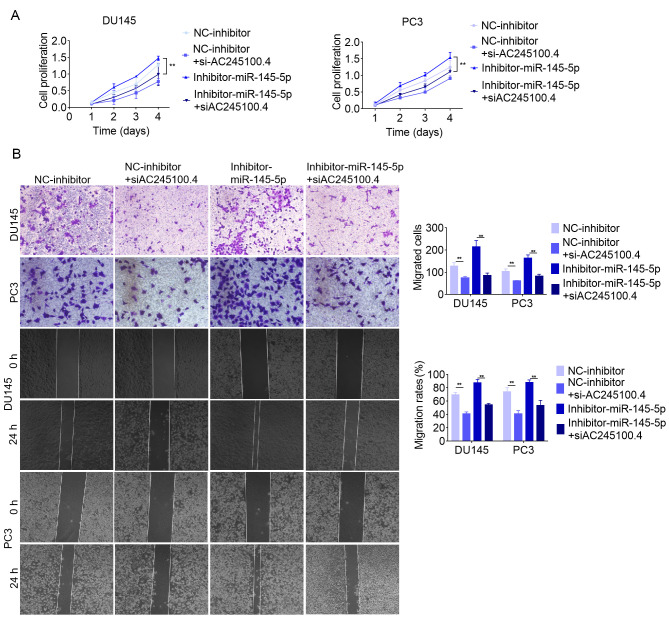
lncRNA AC245100.4 activity is partially mediated via negative regulation of miR-145-5p. (A) Proliferation of DU145 and PC3 cells following co-transfection with si-AC245100.4 and miR-145-5p inhibitor was determined using a Cell Counting Kit 8 assay. (B) Cell migration of DU145 and PC3 cells after co-transfection with si-AC245100.4 and miR-145-5p inhibitor was determined using Transwell migration and wound healing assays. Magnification, ×200. Data are presented as the mean ± SD of three independent experiments (n=3). **P<0.01. lncRNA, long non-coding RNA; miR-145-5p, microRNA-145-5p; si, small interfering RNA; NC, negative control.

